# Preparation and characterization of monoclonal antibodies against porcine gasdermin D protein

**DOI:** 10.1007/s00253-023-12938-x

**Published:** 2024-01-25

**Authors:** Minhui Yang, Xinna Ge, Lei Zhou, Xin Guo, Jun Han, Yongning Zhang, Hanchun Yang

**Affiliations:** 1 National Key Laboratory of Veterinary Public Health and Safety, College of Veterinary Medicine, China Agricultural University, No. 2 Yuanmingyuan West Road, Haidian District, Beijing, 100193 People’s Republic of China; 2 Key Laboratory of Animal Epidemiology of Ministry of Agriculture and Rural Affairs, College of Veterinary Medicine, China Agricultural University, No. 2 Yuanmingyuan West Road, Haidian District, Beijing, 100193 People’s Republic of China

**Keywords:** Pyroptosis, Porcine gasdermin D (pGSDMD), Monoclonal antibodies (mAbs), Epitope, Complementarity-determining region (CDR)

## Abstract

**Abstract:**

Pyroptosis is a newly discovered type of pro-inflammatory programmed cell death that plays a vital role in various processes such as inflammations, immune responses, and pathogen infections. As one of the main executioners of pyroptosis, gasdermin D (GSDMD) is a membrane pore-forming protein that typically exists in a self-inhibitory state. Once activated, GSDMD will be cleaved into an N-terminal fragment with pore-forming activity, becoming the key indicator of pyroptosis activation, and a C-terminal fragment. Although commercial antibodies against human and murine GSDMD proteins are currently available, their reactivity with porcine GSDMD (pGSDMD) is poor, which limits research on the biological functions of pGSDMD and pyroptosis in pigs in vivo and in vitro. Here, five monoclonal antibodies (mAbs) were prepared by immunizing BALB/c mice with procaryotically expressed full-length pGSDMD, all of which did not cross react with human and murine GSDMD proteins. Epitope mapping demonstrated that 15H6 recognizes amino acids (aa) at positions 28–34 of pGSDMD (LQTSDRF), 19H3 recognizes 257–260aa (PPQF), 23H10 and 27A10 recognize 78–82aa (GPFYF), and 25E2 recognizes 429–435aa (PPTLLGS). The affinity constant and isotype of 15H6, 19H3, 23H10, 27A10, and 25E2 mAbs were determined to be 1.32 × 10^−9^, 3.66 × 10^−9^, 9.04 × 10^−9^, 1.83 × 10^−9^, and 8.00 × 10^−8^ mol/L and IgG1/κ, IgG2a/κ, IgG2a/κ, IgG1/κ, and IgG1/κ, respectively. Heavy- and light-chain variable regions sequencing showed that the heavy-chain complementarity-determining region (CDR) sequences of all five mAbs are completely different, while the light-chain CDR sequences of the four mAbs that recognize the N-terminus of pGSDMD are identical. Our prepared mAbs provide valuable materials for studying pGSDMD function and pyroptosis.

**Key points:**

*• A total of five mouse anti-pGSDMD mAbs were prepared, of which four recognize the N-terminus of pGSDMD and one recognize its C-terminus.*

*• The main performance parameters of the five mAbs, including epitope, antibody titer, affinity constant, isotype, and heavy- and light-chain CDR, were characterized.*

*• All five mAbs specifically recognize pGSDMD protein and do not cross react with human and murine GSDMD proteins.*

**Supplementary Information:**

The online version contains supplementary material available at 10.1007/s00253-023-12938-x.

## Introduction

Cell death is a fundamental physiological and pathological process in all living organisms. Its functions cover a wide range from embryonic development, organ maintenance, to the immune response (Bertheloot et al. 2021; Pandian and Kanneganti 2022). As a protective mechanism, cell death helps to eliminate endogenous and exogenous dangers to keep the balance of cell survival and death; maintain the basic functions of various cells, tissues, and organs; and maintain the homeostasis of the whole living body of the organism (Kroemer et al. 2005; Xu et al. 2014). Pyroptosis, also known as “inflammatory necrosis”, is a new type of pro-inflammatory programmed cell death, which ultimately culminates in the loss of plasma membrane integrity (Kroemer et al. 2009). It was first described in 1990s and mistakenly identified as apoptosis due to caspase dependence (Chen et al. 1996; Hilbi et al. 1997; Zychlinsky et al. 1992). Later, the morphological characteristics of this new form of cell death were observed to be quite different from apoptosis, but similar to necrosis instead, causing cell swelling and lysis, though it could be regulated by the signaling cascades via canonical and non-canonical pathways such as apoptosis (Bertheloot et al. 2021; Galluzzi et al. 2018, 2012). As a consequence, scientists began to realize that this is a new type of programmed cell death.

In the canonical pathway, pyroptosis is triggered by intracellular sensors such as NOD‑like receptor family pyrin domain containing 3 (NLRP3) and absent in melanoma 2 (AIM2), by which the cells can detect damage-associated molecular patterns (DAMPs) and pathogen-associated molecular patterns (PAMPs), caused by pathogen infection, pathological injury, and some other stimuli (Sharma and de Alba 2021; Shi et al. 2017; Van Opdenbosch and Lamkanfi 2019). Upon activation, these sensors recruit the adapter ASC (apoptosis-associated speck-like protein containing a CARD), forming an oligomeric structure called the inflammasome, thereby activating the pro-caspase-1, which dimerizes as a pro-form and generates the fully active p20/p10 fragments through autoproteolysis (Liu et al. 2020; Lu et al. 2016; Miao et al. 2010). On the one hand, the activated caspase-1 cleaves the inflammatory cytokines pro-IL-1β and pro-IL-18 to produce mature IL-1β and IL-18 (Hilbi et al. 1997). On the other hand, the mature caspase-1 cleaves gasdermin D (GSDMD) into a N-terminal 31-kDa fragment and a C-terminal 22-kDa fragment, the former of which functions to form pores in the plasma membrane, thereby releasing the mature IL-1β and IL-18, ultimately leading to osmotic imbalance, cell swelling, and even lysis (Shi et al. 2015; Xia et al. 2021).

In recent years, our understanding of pyroptosis has expanded substantially. For example, it has been proven that pyroptosis can also be triggered independent of caspase-1 in the non-canonical pathways. Several pro-inflammatory caspases, such as murine caspase-11 and human caspase-4/-5, can directly target GSDMD for cleavage (Man et al. 2017; Shi et al. 2015). Similarly, other gasdermin family proteins, such as gasdermin A (GSDMA), gasdermin B (GSDMB), gasdermin C (GSDMC), and gasdermin E (GSDME), have also been identified to execute pyroptosis functions similar to GSDMD, which can be cleaved and activated by various proteases (Shao 2021). For instance, GSDMB and GSDME can be cleaved by granzyme A and granzyme B, respectively (Zhang et al. 2020; Zhou et al. 2020). Furthermore, some apoptotic caspases, such as caspase-3 and caspase-8, can also target gasdermins, thus turning apoptosis into pyroptosis (Sarhan et al. 2018; Wang et al. 2017). Consequently, the latest definition suggests that the type of cell death, namely, pyroptosis or apoptosis, does not depend on the pro-inflammatory or apoptotic caspases but rather on the substrate. In other words, pyroptosis is a membrane pore-forming, pro-inflammatory type of programmed cell death mediated by gasdermin family members (Shi et al. 2017). The gasdermin family members share an N-terminal pore-forming domain, an interdomain linker, and a C-terminal autoinhibitory domain (Ding et al. 2016). Some proteases like caspases cleave the interdomain linker in gasdermins, and then, the unleashed N-terminal domain is responsible for penetrating the plasma membrane to cause cell lysis. GSDMD, the charter member of the family, is one of the best studied and is involved in caspase-1- and caspase-11/-4/-5-mediated pyroptosis in canonical and non-canonical pathways, respectively (Wang et al. 2020a). Research shows that GSDMD is widely expressed in different tissues and cell types, with high expression levels in macrophages, monocytes, dendritic cells, and other myeloid cells (Broz et al. 2020). However, so far, there has been relatively little research on porcine GSDMD (pGSDMD) protein compared to human and mouse GSDMD proteins (Wen et al. 2021). Recently, Song and colleagues demonstrated that pGSDMD is a substrate of porcine caspase-1 and an executioner of pyroptosis and that porcine caspase-1 activates pyroptosis by cleaving pGSDMD between aspartic acid (D) 279 and glycine (G) 280 (Song et al. 2022).

As a research frontier hotspot in the field of life sciences in recent years, the functions and mechanisms of pyroptosis have gradually been elucidated. As is well known, the use of GSDMD antibodies is necessary for conducting research on pyroptosis, especially the specific monoclonal antibodies (mAbs). Currently, although commercial antibodies against human and murine GSDMD proteins can be purchased, our research results indicate that their reactivity with pGSDMD protein is poor (Supplemental Fig. S1), which limits the research on the biological functions of pGSDMD and pyroptosis in pigs in vivo and in vitro. So far, there are very few published literatures on the preparation of monoclonal or polyclonal antibodies against pGSDMD protein. Only two research papers published by Song and colleagues mentioned a rabbit anti-pGSDMD polyclonal antibody (Song et al. 2021) and a mouse anti-pGSDMD monoclonal antibody that recognizes the C-terminus of pGSDMD (Song et al. 2022). In the presented study, we successfully prepared five pGSDMD-specific mAbs and systematically characterized them.

## Materials and methods

### Main reagents and antibodies

DNA polymerase (KFX-201) was purchased from TOYOBO Co., Ltd. (Shanghai, China). Adenosine triphosphate (ATP) (HY-B2176) was purchased from MedChemExpress Co., Ltd. (Shanghai, China). Lipopolysaccharide (LPS) (L2630) was purchased from Aldrich Chemical Co., Inc. (Milwaukee, USA). Mouse anti-His-Tag mAb (66,005–1-Ig), rabbit anti-GST-Tag polyclonal antibody (10,000–0-AP), and rabbit anti-GSDMD polyclonal antibody (20,770–1-AP) were purchased from Proteintech Group, Inc. (Wuhan, China). Another rabbit anti-GSDMD polyclonal antibody (A20197) was purchased from ABclonal, Inc. (Wuhan, China). Alexa Fluor™ 488-conjugated goat anti-mouse IgG (H + L) secondary antibody (A-11029), Alexa Fluor™ 568-conjugatd goat anti-mouse F(ab′)2 fragment (A11019), and Lipofectamine LTX (15338100) were purchased from Thermo Fisher Scientific Inc. (Carlsbad, CA, USA). Horseradish peroxidase (HRP)–conjugated goat anti-mouse secondary antibody (ZB-2305) was purchased from ZSGB-BIO Co., Ltd. (Beijing, China). Rabbit anti-pGSDMD polyclonal antibody was prepared in our laboratory using the prokaryotic expressed pGSDMD protein, which will be described in detail below, as the immunogen.

### Vectors, plasmids, and cells

Prokaryotic expression vectors pET30a( +) and pGEX-6p-1 as well as eukaryotic expression vector pCMV-C-HA were preserved in our laboratory. *E*. *coli* DH5α and Rosette (DE3) were purchased from Tsingke Biotechnology Co., Ltd. (Beijing, China). The full-length pGSDMD (pGSDMD-FL) was cloned into the pCMV-C-HA vector to construct a recombinant plasmid pCMV-pGSDMD-FL, which will produce a fusion protein carrying an N-terminal Flag-tag and a C-terminal HA-tag. The N-terminal fragment of pGSDMD (pGSDMD-NT) with pore forming activity was cloned into pCMV-C-HA to construct a recombinant plasmid pCMV-pGSDMD-NT, which will produce a fusion protein carrying an N-terminal Flag-tag. The C-terminal fragment of pGSDMD (pGSDMD-CT) was cloned into pCMV-C-HA to construct a recombinant plasmid pCMV-pGSDMD-CT, which will produce a fusion protein carrying a C-terminal HA-tag. The primers used for construction of these three recombinant eukaryotic plasmids are shown in Supplemental Table S1. Sp2/0-Ag14 (ATCC CRL-1581) and hybridoma cells were cultivated in Dulbecco’s modified Eagle medium (DMEM; ThermoFisher, USA) supplemented with 20% fetal bovine serum (FBS) and 1% penicillin/streptomycin (P/S). Syrian baby hamster kidney cells (BHK-21, ATCC CCL-10) and mouse leukemic monocyte/macrophage cells (RAW264.7, ATCC TIB-71) were cultured in DMEM with 10% FBS and 1% P/S. Porcine lung alveolar macrophage (3D4/21, ATCC CRL-2843) and human monocyte cell line Tohoku Hospital Pediatrics-1 (THP-1, ATCC TIB-202) were cultured in Roswell Park Memorial Institute 1640 medium (ThermoFisher, USA) supplemented with 10% FBS and 1% P/S. All cells were cultured at 37 °C with 5% CO_2_.

### GSDMD gene and protein

The codon of the *Sus scrofa* GSDMD gene (GenBank accession no. XM_021090506.1) was optimized for optimal usage in *Escherichia coli* (*E*. *coli*) using GenSmart™ Codon Optimization software. The optimized sequence (Supplemental File S1) was synthesized by Beijing Protein Innovation Co., Ltd. (Beijing, China), and then cloned into the pET30a( +) vector between *Eco*R I and *Xho* I sites to generate the recombinant plasmid pET30a( +)-pGSDMD. The plasmid was sequenced by Sanger sequencing to ensure its accuracy (data not shown). Furthermore, *Homo sapiens* GSDMD (NP_001159709.1), *Mus musculus* GSDMD (NP_081236.1), *Rattus norvegicus* GSDMD (NP_001387923.1), *Pan troglodytes* GSDMD (XP_009454389.3), *Bos taurus* GSDMD (NP_001346905.1), and *Capra hircus* GSDMD (ALN66870.1) were subjected to amino acid sequence alignment using MEGA-X and DNASTAR softwares.

### Structure prediction and bioinformatic analysis of pGSDMD

The 3-D structure and secondary structure of pGSDMD protein were predicted by SWISS-MODEL (https://swissmodel.expasy.org/) and Phyre^2^ (http://www.sbg.bio.ic.ac.uk/phyre2/html/page.cgi?id=index) online web tools, respectively. Subsequently, the pGSDMD protein structure was labeled with PyMOL. In order to identify the transmembrane, intracellular, and extracellular domains, the amino acid sequence of pGSDMD was analyzed by the TMHMM Server v.2.0 online web tool (http://www.cbs.dtu.dk/services/TMHMM-2.0/). Moreover, Immune Epitope Database and Analysis Resource (IEDB) online bioinformatic software (http://tools.immuneepitope.org/bcell/result/) was used to predict the B-cell epitope of pGSDMD.

### Expression of recombinant pGSDMD protein

The recombinant plasmid pET30a( +)-pGSDMD was transformed into *E*. *coli* Rosette (DE3) competent cells and cultured at 37 °C overnight. A single bacterial colony was picked and inoculated into Luria–Bertani (LB) medium supplemented with 1% kanamycin. After overnight culture at 37 °C, 1 mL culture was added to 150 mL fresh LB medium and incubated at 200 rpm for about 3 h until the optical density at 600 nm wavelength of the culture reached 0.6–0.8. Isopropyl β-D-thiogalactopyranoside (IPTG) was added to a final concentration of 0.5 mM to induce protein expression. The empty vector pET30a( +) was treated in the same way and used as a control. After culture at 37 °C for 4 h, the culture was centrifuged for 10 min at 3000 g. The supernatants were discarded, and the pellets were resuspended with pH 7.4 phosphate-buffered saline (PBS). After ultrasonication (400 w, 3-s work, 5-s interval) for 5 min in ice bath, the lysates were centrifuged at 3000 g for 15 min at 4 °C. Both supernatants and precipitates were used for the subsequent soluble analysis. The expression of recombinant pGSDMD protein was determined by sodium dodecyl sulfate polyacrylamide gel electrophoresis (SDS-PAGE), followed by Coomassie brilliant blue staining.

### Purification of recombinant pGSDMD protein

Since the recombinant pGSDMD protein was mainly expressed in insoluble aggregates, inclusion bodies were isolated and subjected to denaturation and renaturation, followed by nickel-nitrilotriacetic acid (Ni–NTA) agarose (Qiagen, Hilden, Germany) affinity chromatography purification according to the manufacturer’s instructions. Briefly, the recombinant pGSDMD protein was incubated with the Ni–NTA agarose for 1 h at 4 °C. After washing with wash buffer containing 8 M urea to remove the impurities, pGSDMD was eluted with elution buffer containing 8 M urea and 500 mM imidazole. Afterwards, the purified protein solutions were concentrated through polyethylene glycol 20,000 and then analyzed by SDS-PAGE.

### Indirect enzyme-linked immunosorbent assay (iELISA)

Ninety-six ELISA plates were coated with 100 ng/well of purified pGSDMD protein diluted in 100 μL pH 9.5 carbonate buffer solution. The plates were incubated at 37 °C for 3 h. After washing five times with PBST (PBS containing 0.05% Tween-20), 100 μL/well of 5% skim milk diluted in PBST was added to block the wells for 2 h at 37 °C. After another washing step, 100 μL/well of hybridoma cell culture supernatants or diluted mAbs was added to the plates, along with positive control (serum of the hyperimmune mouse), negative control (serum of the non-immunized mouse), and blank control. The plates were incubated at 37 °C for 1 h. After washing five times with PBST, 100 μL/well of HRP-conjugated goat anti-mouse secondary antibody (ZSGB-BIO, China) at 1:5000 dilution was added to the plates and incubated at 37 °C for 30 min. After a final wash, 100 µL/well of tetramethylbenzidine (TMB) substrate (Solarbio, China) was added to the plates for a 10-min chromogenic reaction at room temperature in the dark. The chromogenic reaction was stopped by adding 50 µL/well of 5M H_2_SO_4_. The optical density at 450 nm (OD_450nm_) was determined using a microplate reader (TECAN, Switzerland). The testing results were considered positive for anti-pGSDMD antibodies if the OD_450nm_ value was 2.1-fold higher than that of the negative control.

### Preparation of mAbs against pGSDMD

Mouse anti-pGSDMD mAbs were prepared as previously described with a slight modification (Zhang et al. 2013). Briefly, BALB/c mice were immunized subcutaneously with 60 μg of purified pGSDMD emulsified in Freund’s complete adjuvant (Sigma-Aldrich, USA). Two weeks later, the mice were successively boosted thrice with 30 μg of pGSDMD emulsified in Freund’s incomplete adjuvant at 2-week intervals. Three days after the last booster immunization, the splenocytes of immunized mice were processed for fusion with Sp2/0 cells. The generating positive hybridoma cells were selected by an indirect ELISA using the purified pGSDMD as the coating antigen. The hybridoma cells whose supernatants positively reacted with pGSDMD protein were subjected to three rounds of subcloning.

#### SDS-PAGE

Protein samples were boiled with 5 × loading buffer for 6 min at 95 °C. Approximately 40 mg/lane of each boiled sample was fractionated by electrophoresis on 12.5% SDS-PAGE gels. After electrophoresis, the gels were stained with Coomassie Blue Fast Staining Solution (Solarbio, China) for 10 min. Digital images of the gels were taken using a Bio-Rad Gel Imager System (Hercules, CA, USA).

### Western blot

The proteins were transferred from the gel to polyvinylidene fluoride (PVDF) membranes using a wet-transfer method at 100 V 120 min. After blocking with 5% skim milk for 2 h at room temperature, the membrane was incubated with the primary antibodies for 2 h at room temperature. After washing five times with PBST, the membrane was incubated with corresponding secondary antibodies (goat anti-mouse or anti-rabbit IgG) conjugated to HRP (ZSGB-BIO, China). After final washing with PBST, the membrane was treated with enhanced chemiluminescence reagents (Engreen Biosystem Co, Ltd, Beijing, China) and target proteins were visualized using a Bio-Rad Gel Imager System.

### Titer determination and isotype identification of mAbs

For mAb titer determination, a series of twofold dilutions of each mAb ranging from 1:200 to 1:204,800 were determined by the aforementioned iELISA. For mAb isotype identification, the prepared mAbs were determined using a mouse mAb isotyping ELISA kit as per the manufacturer’s instructions (SouthernBiotech, Birmingham, AL, USA).

### Determination of heavy- and light-chain variable regions of mAbs

The variable regions of heavy and light chains of each mAb were amplified by polymerase chain reaction (PCR) using the primers listed in Supplemental Table S2 (Jia et al. 2022). Each amplicon containing the variable regions was cloned into the *pEASY*-Blunt Zero vector (TransGen, Beijing, China). The resulting positive recombinant plasmids were sequenced by Tsingke Biotechnology Co., Ltd. (Beijing, China) using the vector universal primers (M13F/R). Subsequently, the sequencing results were subjected to annotation analysis of heavy-chain variable regions (CDR-H1, CDR-H2, and CDR-H3) and light-chain variable regions (CDR-L1, CDR-L2, and CDR-L3) using the Kabat antibody numbering scheme (https://www.novopro.cn/tools/cdr.html) online web tool.

### Determination of mAb affinity constant

The aforementioned iELISA was used to determine the affinity constant of each mAb using the serial twofold dilutions ranging from 1:200 to 1:204,800. On the basis of completing the OD_450nm_ value measurement, scatter plots were plotted using the OD_450nm_ values against the mAb concentrations. Subsequently, the fitting curves were drawn by Origin 8.0 software (OriginLab, MA, USA), using mAb concentrations as the *X*-axis and OD_450nm_ values as the *Y*-axis. Half of the maximum OD_450nm_ value in the iELISA tests was imported into the fitting curve to calculate the corresponding mAb concentration, which was defined as the affinity constant of each mAb.

### Immunofluorescent assay (IFA)

The constructed three plasmids, pGSDMD-FL, pGSDMD-NT, and pGSDMD-CT, were separately transfected into BHK-21 cells using Lipofectamine LTX reagent (ThermoFisher, USA) according to the manufacturer’s instructions. At 24 h post-transfection, the cells were fixed with 4% paraformaldehyde for 10 min and then permeabilized with 0.1% triton X-100 for 10 min. After blocking with 2% BSA for 30 min, the cells were probed with each of the mAbs at 1:1000 dilution for 2 h at room temperature. After washing five times with PBST, the cells were incubated with Alexa Flour™ 488-conjugated goat anti-mouse secondary antibody (ThermoFisher, USA) for 1 h at room temperature. After washing five times with PBST, the cells were counterstained with DAPI (Solarbio, China). After a final washing step, images of the cells were taken with a fluorescence microscope (Nikon, Japan).

### Epitope mapping of the pGSDMD mAbs

To map the B-cell epitope recognized by each mAb, truncated forms of pGSDMD gene were separately cloned into vector pGEX-6p-1 to generate a series of adjacent truncations overlapping with each other. Briefly, all pGSDMD truncated fragments were amplified by PCR using plasmid pET30a( +)-pGSDMD as the template and with primers listed in Supplemental Table S3. The amplified fragments corresponding to each truncation were separately cloned into vector pGEX-6p-1 to generate recombinant plasmids. After sequencing to ensure accuracy, the plasmids were transformed into *E. coli* Rosette (DE3) and induced with 0.5 M IPTG at 37 °C for 4 h to express GST-tagged truncated pGSDMD fusion proteins. The truncated pGSDMD proteins were subjected to western blot analysis using rabbit anti-GST polyclonal antibody and the prepared mAbs as the primary antibodies, respectively.

### Assessment of mAb specificity

Three representative cells RAW264.7, THP-1, and 3D4/21 from distinct species of *Mus musculus*, *Homo sapiens*, and *Sus scrofa*, respectively, were used to evaluate the specificity of the five mAbs raised against pGSDMD protein. To this end, the cells were treated without or with LPS plus ATP (primed with 1 μg/mL LPS for 3 h and then stimulated with 10 mM ATP for 1 h), which has been widely used as a positive control for pyroptosis activation (Gao et al. 2022; Seoane et al. 2020). Subsequently, the treated cells and the control cells were lysed with RIPA buffer (Beyotime, China) supplemented with protein inhibitors (Roche, Switzerland) for 30 min in ice bath. The cell lysates were subjected to western blot analyses using the prepared five mAbs or two commercially available polyclonal antibodies as the primary antibodies.

### Statistical analysis

Student’ s *t*-test with two-tailed distribution and two sample unequal variance were performed to analyze the statistical significance of two comparison. The data were analyzed and processed by GraphPad Prism version 8.0 software (La Jolla, CA, USA).

## Results

### Bioinformatic analysis, prokaryotic expression, and purification of pGSDMD protein

**Fig. 1:**
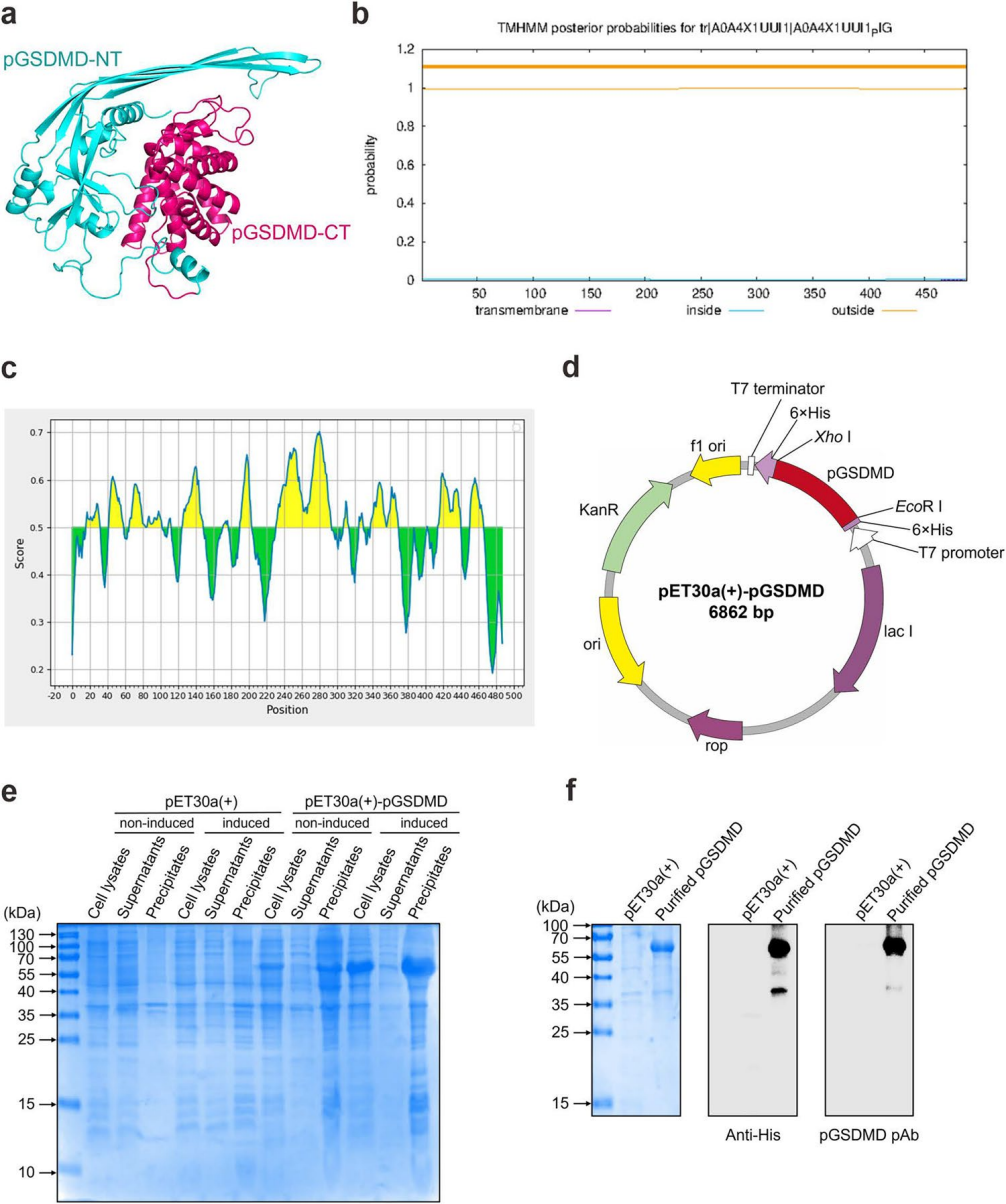
Bioinformatic analysis, prokaryotic expression, and purification of pGSDMD protein. **a** 3-D structure prediction of pGSDMD by SWISS-MODEL. **b** Transmembrane region prediction of pGSDMD by TMHMM. **c** B-cell epitope prediction of pGSDMD by IEDB. **d** Construction strategy of the prokaryotic expression plasmid pET30a( +)-pGSDMD. **e** SDS-PAGE analysis of the solubility of recombinant pGSDMD protein expressed in *E. coli* Rosette (DE3) competent cells. **f** SDS-PAGE analysis and western blot identification of the purified pGSDMD protein using mouse anti-His tag antibody and rabbit anti-pGSDMD polyclonal antibody (pAb)

SWISS-MODEL prediction showed that pGSDMD protein exhibits a two-domain architecture composed of a spherical pGSDMD-CT domain and an extended twisted pGSDMD-NT domain (Fig. 1a). When GSDMD is in a resting state, pGSDMD-NT interacts with pGSDMD-CT to form the same automatic inhibitory structure as human and murine GSDMD proteins (Ding et al. 2016). Moreover, Phyre^2^ predicted that the secondary structure of pGSDMD protein includes 34% hydrophobic α-helix (Fig. 1a and Supplemental Fig. S2), which means that the solubility of pGSDMD protein is poor. In general, membrane proteins are difficult to express in prokaryotic expression systems, and deleting their transmembrane regions can aid in the expression of such proteins. For this reason, we predicted pGSDMD using TMHMM Server v.2.0 and found that there are no recognizable transmembrane and inside regions in the pGSDMD protein (Fig. 1b). Furthermore, B-cell epitope prediction of pGSDMD by IEDB showed that the antigenicity of pGSDMD is excellent (Fig. 1c). On the basis of above bioinformatic analysis results, we decided to express the full-length pGSDMD protein using the constructed prokaryotic expression plasmid pET30a( +)-pGSDMD (Fig. 1d).

### Expression and purification of recombinant pGSDMD protein

Following SDS-PAGE analysis and Coomassie brilliant blue staining, a thick protein band with a molecular weight around 60 kDa (Fig. 1e) was only detected in *E. coli* Rosette (DE3) cells transformed with pET30a( +)-pGSDMD after induction with IPTG. The size of the protein band corresponds to the theoretical molecular weight calculation for the recombinant pGSDMD protein. Notably, pGSDMD protein was mainly expressed in the form of inclusion bodies under the conditions we used (0.5 mM IPTG, 37 °C 4 h). Hence, the precipitates of whole-cell lysates were subjected to protein purification using Ni–NTA agarose. After purification, a clear protein band with a molecular weight of approximately 60 kDa was detected (Fig. 1f). The concentration of the purified pGSDMD protein was determined to be 0.85 mg/mL. Western blot analysis further showed that the purified pGSDMD protein could be recognized by both mouse anti-His-tag mAb and rabbit anti-pGSDMD polyclonal antibody (Fig. 1f).

### Preparation and features of mouse anti-pGSDMD mAbs

**Fig. 2:**
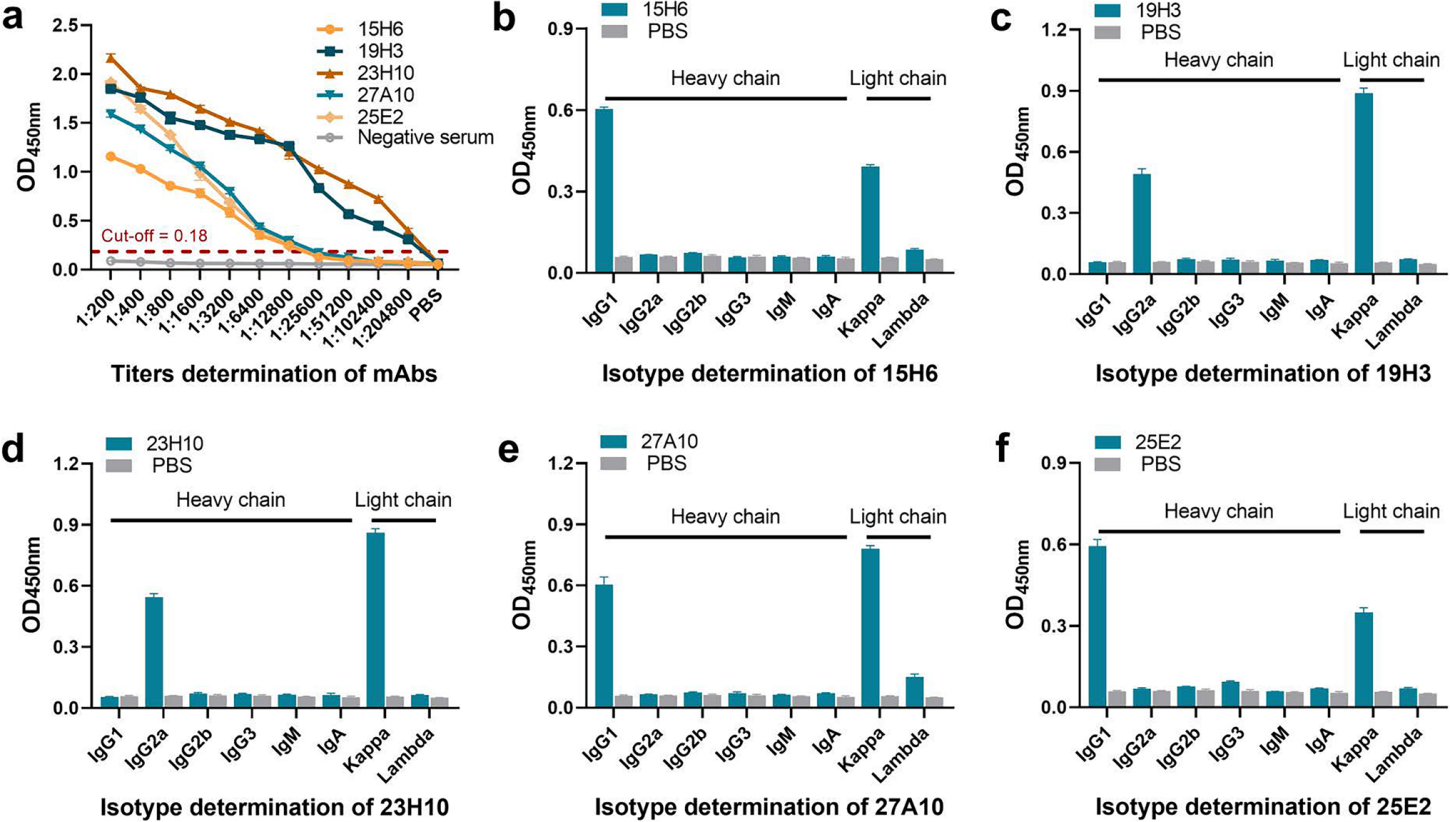
Titer determination and isotype identification of the prepared mouse anti-pGSDMD mAbs. **a** The titers of the five mAbs were determined by an indirect ELISA. **b**–**f** The isotypes of the five mAbs were determined by a mouse mAb isotyping ELISA kit

Through iELISA screening, a total of five hybridoma cell clones stably secreting anti-pGSDMD mAbs were obtained. HiTrap rProtein A FF columns (GE Healthcare, WI, USA) were used to purify mAbs from the ascites of BALB/c mice intraperitoneally injected with the obtained hybridoma cells. The titer of the purified mAbs 15H6, 19H3, 23H10, 27A10, and 25E2 was determined to be 1:12,800, 1:204,800, 1:204,800, 1:12,800, and 1:12,800, respectively (Fig. 2a). The isotype of mAbs 15H6, 19H3, 23H10, 27A10, and 25E2 was determined to be IgG1/κ, IgG2a/κ, IgG2a/κ, IgG1/κ, and IgG1/κ, respectively (Fig. 2b–f).

### Sequencing of heavy- and light-chain variable regions of pGSDMD mAbs

**Fig. 3:**
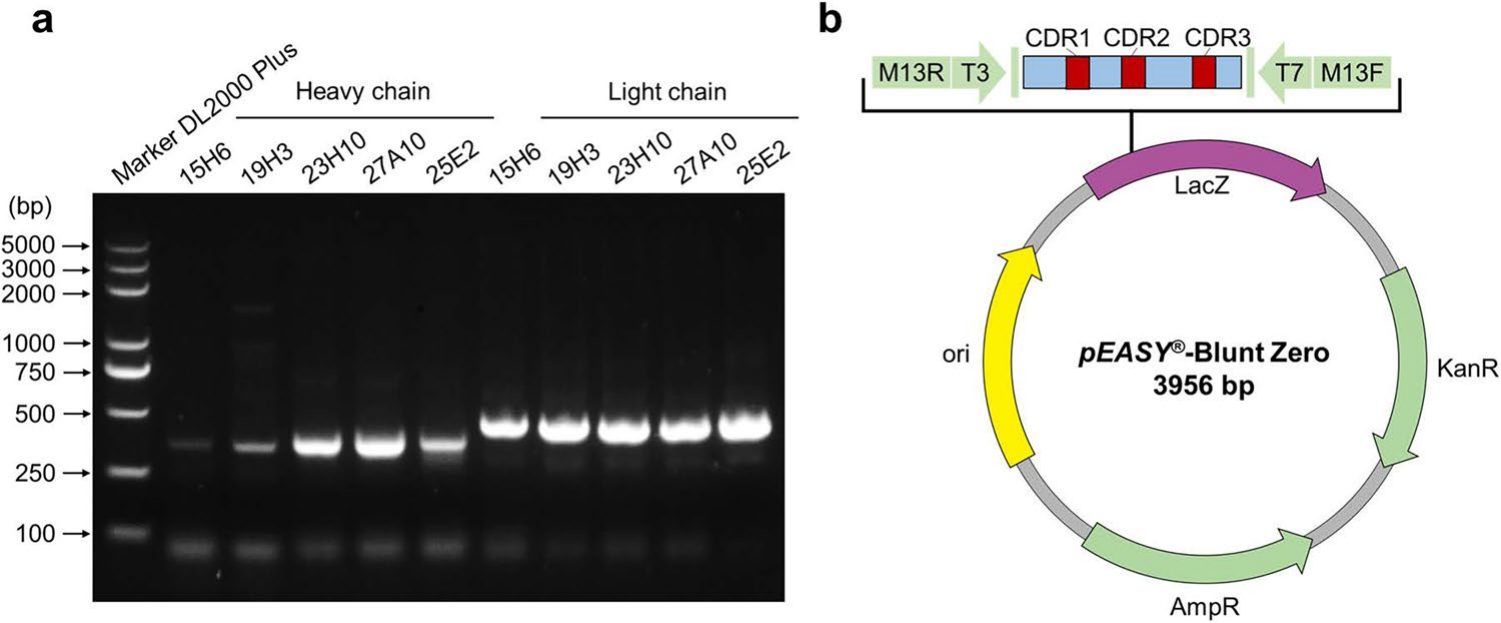
PCR amplification of the heavy- and light-chain variable regions of the prepared mouse anti-pGSDMD mAbs and the construction strategy of recombinant plasmids for sequencing. **a** PCR amplification results of the heavy- and light-chain variable regions of the five pGSDMD mAbs. **b** Construction strategy of recombinant plasmids for sequencing using *pEASY*-Blunt Zero vector. M13F and M13R are universal sequencing primers of the vector. CDR complementarity-determining region

After PCR amplification, the amplicons of the heavy- and light-chain variable regions of each mAb were cloned into the *pEASY*-Blunt Zero vector for sequencing (Fig. 3a, b). The obtained nucleotide sequences of the heavy and light chains of each mAb were subjected to the NCBI database under accession numbers OR468141‒OR468146 and OR482587‒OR482590 (Table 1), and their corresponding amino acid sequences were analyzed using the Kabat antibody numbering scheme to annotate the CDR-H1, CDR-H2, CDR-H3, CDR-L1, CDR-L2, and CDR-L3 of each mAb. As shown in Table 1, the amino acid sequences of the heavy-chain CDR region of the five mAbs are different from each other, while the amino acid sequences of the light-chain CDR region of the four mAbs (15H6, 19H3, 23H10, and 27A10) recognizing GSDMD-NT are identical.

**Table 1:** Amino acid sequences of the heavy- and light-chain variable regions of the five mouse anti-pGSDMD mAbs

mAbs name	Variable region of heavy chain	Variable region of light chain	NCBI accession number
15H6	RTSAWREVKISCKASGYTFTDYSIHWVNQAPGKGLQWMGWINTATHEPKYADDFKGRFAFSLETSASTAYLQINNLKNEDTSTYFCTRDAYYWYFDVWGAGTMVPSPQKRAKARLILIFRTNTDRESGASRSF CDR-H1: DYSIH CDR-H2: WINTATHEPKYADDFKG CDR-H3: DAYYWYFDV	ASLAVSLGQRATISYRASKSVSTSGYSYMHWNQQKPGQPPRLLIYLVSNLESGVPARFSGSGSGTDFTLNIHPVEEEDAATYYCQHIRELTRSEGGPSWK CDR-L1: RASKSVSTSGYSYMH CDR-L2: LVSNLES CDR-L3: QHIR	Heavy chain: OR468144 Light chain: OR468142
19H3	MTMITPSSELTLTKGTSPAGLNELALEVKLLESGGGLAQPGGSMKLSCVSSGFAFSTSWMSWVRQSPEKGLEWVAEVRLKSDNYAKHYAESVKGRFTISRDDSTSRLTLQMNSLRAEDTGIYYCTDLINWGQGTMVTVS CDR-H1: TSWMS CDR-H2: EVRLKSDNYAKHYAESVKG CDR-H3: LIN	ASLAVSLGQRATISYRASKSVSTSGYSYMHWNQQKPGQPPRLLIYLVSNLESGVPARFSGSGSGTDFTLNIHPVEEEDAATYYCQHIRELTRSEGGPSWK CDR-L1: RASKSVSTSGYSYMH CDR-L2: LVSNLES CDR-L3: QHIR	Heavy chain: OR482587 Light chain: OR468146
23H10	MSCKASGYTFTTYWIHWIKQTPGQGLEWIGFINPATGYTEYNQKFKDKATLTSDSSSTTAYMQLSSLTSEDSAVYYCAAYYRDDGGDYWGQGTTVPVSSKQS CDR-H1: TYWIH CDR-H2: FINPATGYTEYNQKFKD CDR-H3: YYRDDGGDY	ASLAVSLGQRATISYRASKSVSTSGYSYMHWNQQKPGQPPRLLIYLVSNLESGVPARFSGSGSGTDFTLNIHPVEEEDAATYYCQHIRELTRSEGGPSWK CDR-L1: RASKSVSTSGYSYMH CDR-L2: LVSNLES CDR-L3: QHIR	Heavy chain: OR482588 Light chain: OR468143
27A10	MSCKGSGYTFGNYWMHWVKQRPGQGLEWIGYINPSTGYTEYNVKFKDKATLTADKSSSAVYMQLRSLTSEDSAVYYCANYSGSTYWFASWGQGTTVTVSSKGQFV CDR-H1: NYWMH CDR-H2: YINPSTGYTEYNVKFKD CDR-H3: YSGSTYWFAS	ASLAVSLGQRATISYRASKSVSTSGYSYMHWNQQKPGQPPRLLIYLVSNLESGVPARFSGSGSGTDFTLNIHPVEEEDAATYYCQHIRELTRSEGGPSWK CDR-L1: RASKSVSTSGYSYMH CDR-L2: LVSNLES CDR-L3: QHIR	Heavy chain: OR482589 Light chain: OR468145
25E2	MSCKASGYTFTSYWIHWIKQRPGQGLEWIGAINPGNSDTAYNQKFKGQAKLTAVTSTSTSFMEFSSLTNEDSAVYHCTRSGITGKGGFDYWGQGTTVTVSFKGPIRGR CDR-H1: SYWIH CDR-H2: AINPGNSDTAYNQKFKG CDR-H3: SGITGKGGFDY	MNWFQQKPGQPPKLLIYGASNQESGVPARFSGSGSGTDFSLNIHPMEEDDTAMYFCQQSKEVPWTFGGGTKLEIKRADAAPTVSISHLQK CDR-L1: WFQQKPGQPPKLLIY CDR-L2: GVPARFSGSGSGTDFSLNIHPMEEDDTAMYFC CDR-L3: FGGGTKLEIK	Heavy chain: OR482590 Light chain: OR468141

### Affinity constant of the pGSDMD mAbs

By determining the OD_450nm_ value of serial mAb dilutions with an iELISA and drawing the fitted curves between the OD_450nm_ value and the mAb concentration (Supplemental Fig. S3), the affinity constant of 15H6, 19H3, 23H10, 27A10, and 25E2 was calculated as 1.32 × 10^−9^, 3.66 × 10^−9^, 9.04 × 10^−9^, 1.83 × 10^−9^, and 8.00 × 10^−8^, respectively, which means that all five mAbs have a prominent affinity for pGSDMD protein.

### Reactivity of the five mAbs with pGSDMD-FL, pGSDMD-NT, and pGSDMD-CT

**Fig. 4:**
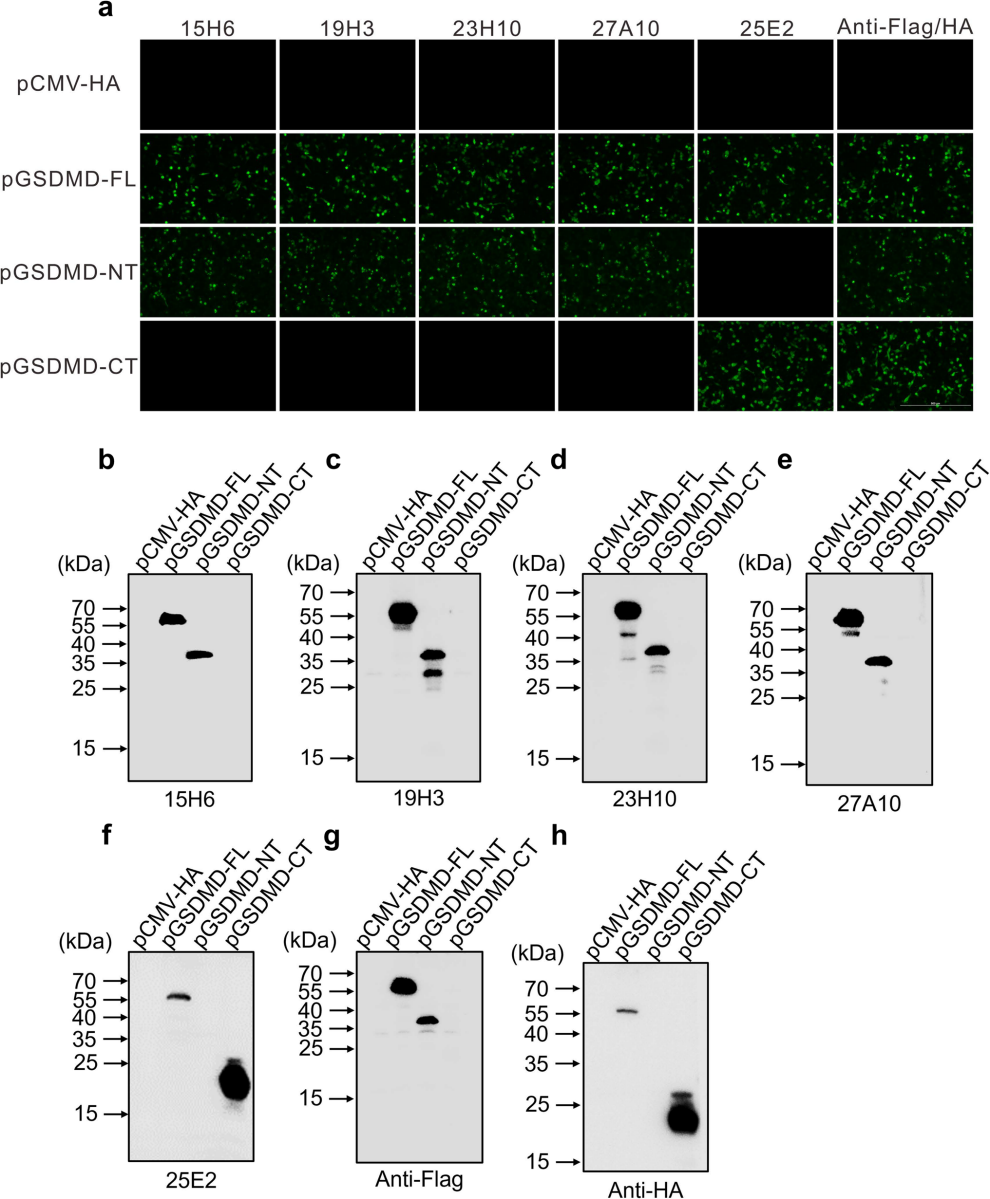
Analysis of the reactivity of the prepared mAbs with pGSDMD-FL, pGSDMD NT, and pGSDMD-CT. **a** IFA analysis of the reactivity of the five mAbs (15H6, 19H3, 23H10, 27A10, and 25E2) with pGSDMD-FL, pGSDMD NT, and pGSDMD-CT, which were expressed in BHK-21 cells by transfection with recombinant eukaryotic plasmids pCMV-pGSDMD-FL, pCMV-pGSDMD-NT, and pCMV-pGSDMD-CT, respectively. The cells transfected with empty vetor pCMV-HA were used as a negative control. **b**–**h** Western blot analysis of the reactivity of the five mAbs with pGSDMD-FL, pGSDMD-NT, and pGSDMD-CT using the cell lysates of BHK-21 transfected as in **a**. The Flag and HA tags were used to indicate the successful cell transfection

The IFA results showed that all five prepared mAbs are able to react with the pGSDMD-FL protein expressed in BHK-21 cells (Fig. 4a). Among them, 15H6, 19H3, 23H10, and 27A10 can further recognize the GSDMD-NT fragment that has membrane pore-forming activity, while 25E2 can recognize the GSDMD-CT fragment (Fig. 4a). These results were further confirmed by the western blot results (Fig. 4b–h). Since protein-denaturing polyacrylamide gel electrophoresis was used to detect the reactivity of the mAbs with pGSDMD protein by western blot, we concluded that the epitopes recognized by the five mAbs are linear.

### B-cell epitopes of the pGSDMD mAbs

**Fig. 5:**
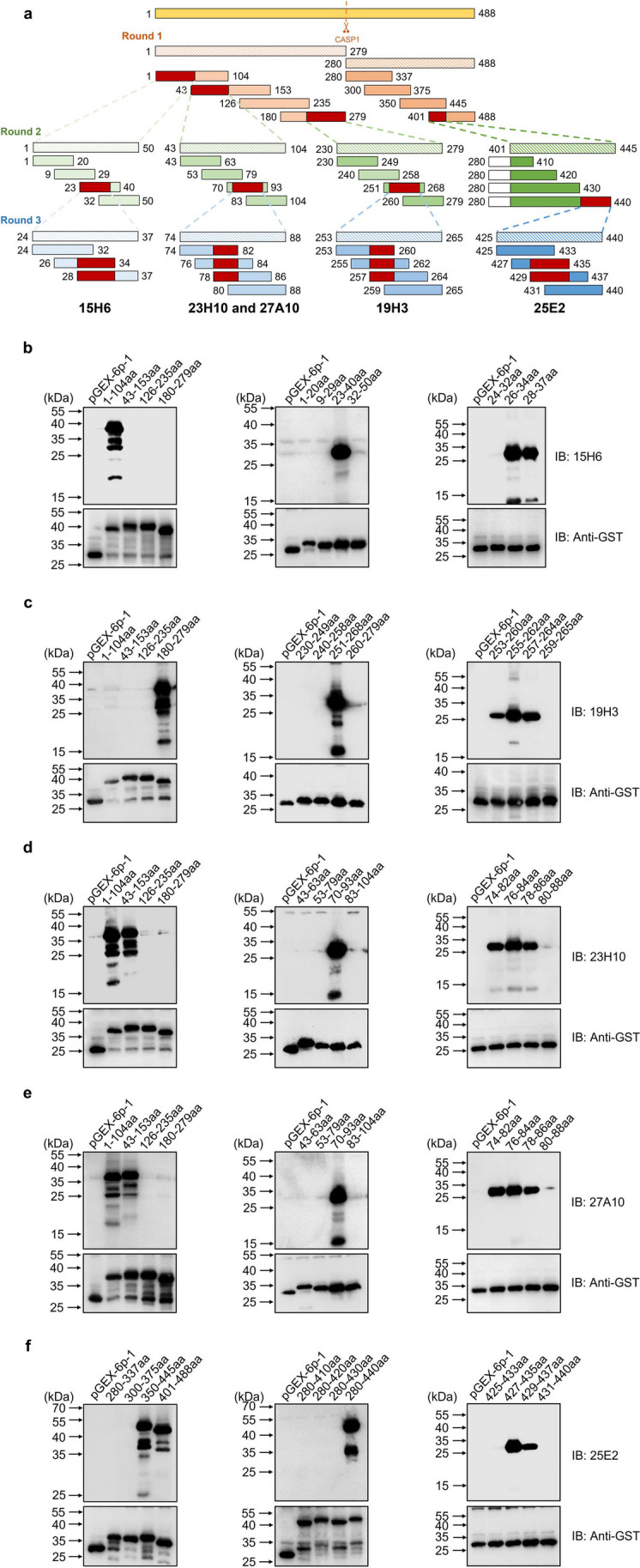
B-cell epitope mapping of the prepared mouse anti-pGSDMD mAbs. **a** Construction strategy of three-round truncated forms of GST-tagged pGSDMD protein. The numbers indicate the amino acid positions in the full-length pGSDMD protein. **b**–**f** Western blot identification of the reactivity of mAbs 15H6, 19H3, 23H10, 27A10, and 25E2 with the truncated GST-tagged pGSDMD, respectively

Three-round construction of gradually fined truncations of GST-tagged pGSDMD (Fig. 5a) was applied to localize the epitopes recognized by the five pGSDMD mAbs using western blot analyses. In the first round of identification, 15H6 recognized the fragment containing 1–104aa, 19H3 recognized the 180–279aa fragment, 23H10 and 27A10 recognized both 1–104aa and 43–153aa fragments, and 25E2 recognized both 350–445aa and 401–488aa fragments (Fig. 5b–f). In the second round of identification, 15H6 recognized 23–40aa fragment, 19H3 recognized 251–268aa fragment, 23H10 and 27A10 recognized 70–93aa fragment, and 25E2 recognized 280–440aa fragment (Fig. 5b–f). In the last round of identification, 15H6 reacted with two fragments containing 28–34aa (LQTSDRF), 19H3 reacted with three fragments containing 257–260aa (PPQF), 23H10 and 27A10 reacted with three fragments containing 78–82aa (GPFYF), and 25E2 reacted with two fragments containing 429–435aa (PPTLLGS). The detailed information of the epitopes recognized by the five pGSDMD mAbs is summarized in Table 2.

**Table 2:** B-cell epitope information of the five mouse anti-pGSDMD mAbs

mAb name	Epitope type	Recognition region	Epitope location	Amino acid
15H6	Linear epitope	pGSDMD-NT	28–34aa	LQTSDRF
19H3	Linear epitope	pGSDMD-NT	257–260aa	PPQF
23H10	Linear epitope	pGSDMD-NT	78–82aa	GPFYF
27A10	Linear epitope	pGSDMD-NT	78–82aa	GPFYF
25E2	Linear epitope	pGSDMD-CT	429–435aa	PPTLLGS

### Specificity assessment of the pGSDMD mAbs

**Fig. 6:**
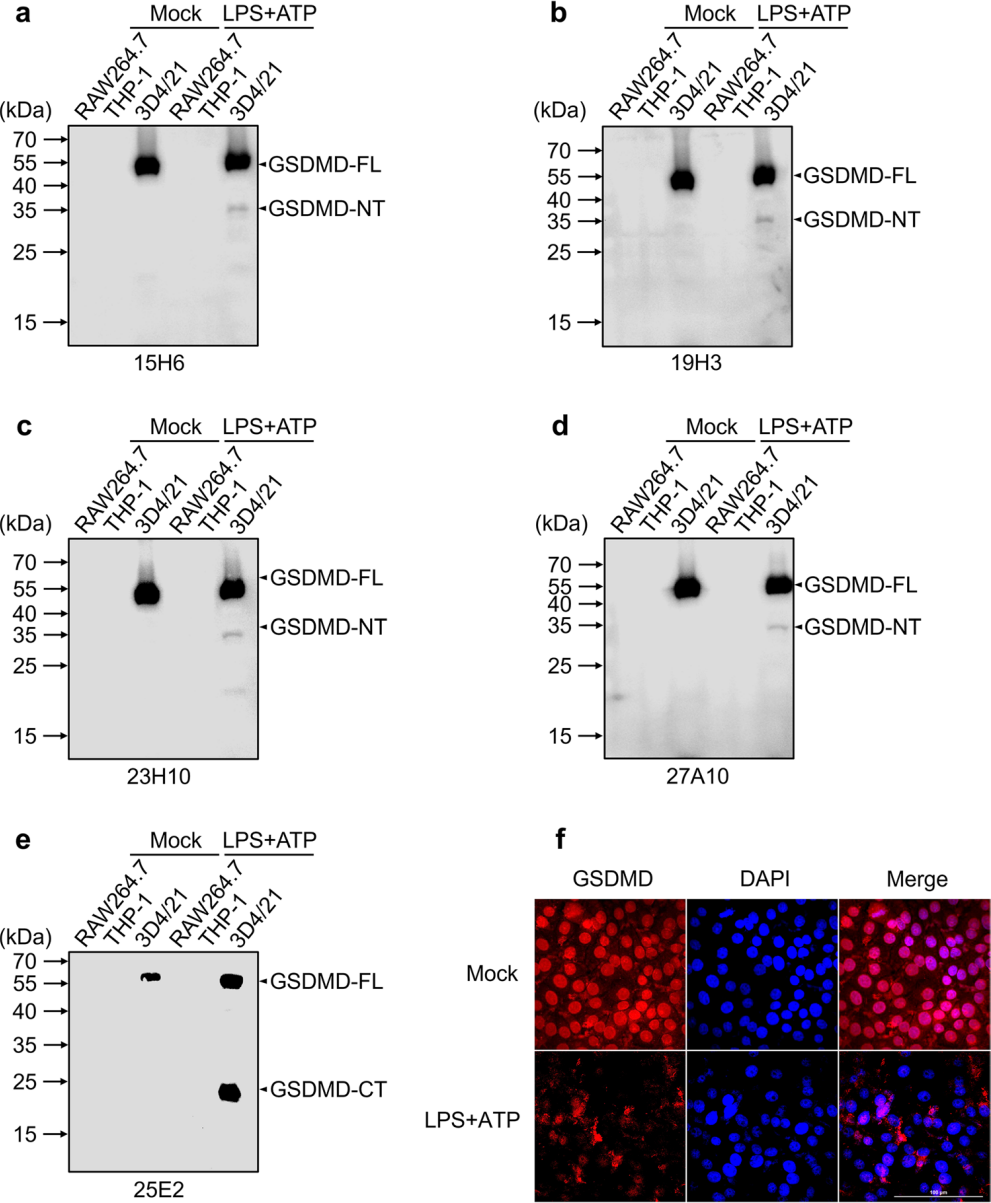
Immunoreactivity analysis of the prepared mouse anti-pGSDMD mAbs with endogenous pGSDMD protein in three cell lines from different species using western blot and IFA assays. **a**–**e** Specificity assessment results of mAbs 15H6, 19H3, 23H10, 27A10, and 25E2, respectively. **f** Representative IFA images of the reactivity of mAb 23H10 with endogenous pGSDMD protein in 3D4/21 cells

In RAW264.7, THP-1, and 3D4/21 cells representing *Mus musculus*, *Homo sapiens*, and *Sus scrofa* species, respectively, the five mAbs only reacted with the endogenous pGSDMD protein in 3D4/21 cells, regardless of whether the pyroptosis in the cells was activated or not (Fig. 6a–e). This indicates that these five mAbs can specifically recognize the pGSDMD protein. In addition, our results further confirmed that mAbs 15H6, 19H3, 23H10, and 27A10 recognize the GSDMD-NT fragment, and 25E2 recognizes the GSDMD-CT fragment (Fig. 6a–e). In addition, we further analyzed the reactivity of the five mAbs with endogenous pGSDMD protein in 3D4/21 cells using IFA assays. Interestingly, among the five mAbs, only 23H10 was able to detect endogenous pGSDMD protein well in the IFA assays (Fig. 6f). Specifically, the immunostaining fluorescent signals of pGSDMD protein were diffusely distributed in the nucleus and cytoplasm of normal (mock treatment) 3D4/21 cells. However, after activating pyroptosis in 3D4/21 cells using LPS + ATP treatment, the pGSDMD immunostaining fluorescent signals developed a distinctly punctate perinuclear distribution (Fig. 6f).

### Conservative analysis of epitopes recognized by mAbs

To analyze the conservation of antigenic epitopes recognized by the five mAbs we prepared (15H6, 19H3, 23H10, 27A10, and 25E2), the only three amino acid sequences of human, mouse, and porcine GSDMD proteins currently available in the GenBank database were used for the alignment. The results showed that the epitopes recognized by these five mAbs are not conserved between human, mouse, and porcine GSDMD proteins (Supplemental Fig. S4). Due to the limited sequence of human, mouse, and porcine GSDMD proteins in the GenBank database, our current study was unable to determine whether there exist common epitopes between GSDMD proteins of different species.

## Discussion

Pigs are not only very important economic animals worldwide but also potential donors for treating certain organic diseases in humans through organ or tissue transplantation (Längin et al. 2018). Therefore, ensuring the health of pig bodies, especially donor pigs, will be of great significance. However, when pigs are invaded by risk factors, such as pathogen infections, their immune system will be activated, leading to complex immune responses in order to fight against the risk factors. However, in some cases, the strong immune response of the body, such as cytokine storm or inflammation storm, will usually cause fatal consequences to pigs (Wang et al. 2020b). For example, African swine fever virus (ASFV) infection, which seriously endangers the global pig farming industry, can lead to widespread bleeding of pig skin, various tissues, and organs, as well as severe lymphoid tissue damage and inflammatory reactions (Dixon et al. 2019). It was reported that various programmed cell deaths, including apoptosis, necrosis, and so on, may be closely related to such pathological damage (Dixon et al. 2019; Salguero 2020). Undoubtedly, revealing the underlying regulatory mechanisms of dangerous immune responses that can be life-threatening in pigs, such as severe inflammatory reactions, can provide new ideas or therapeutic targets for the prevention and control of swine infectious diseases.

In the near decade, a newly defined type of programmed cell death, termed pyroptosis, has been proven to play a crucial role in the pro-inflammatory cell death of humans and mice, which is executed by the gasdermin family proteins with membrane pore-forming activity (Pandian and Kanneganti 2022). One of these proteins, GSDMD, has been identified as the main executioner of pyroptosis and has become a research hotspot in various human and murine cell death studies. Although multiple commercial antibodies against human and murine GSDMD proteins are currently available, these antibodies do not react or have poor reactivity with pGSDMD protein (Supplemental Fig. S1). Nonetheless, Xu and colleagues demonstrated that a commercially available rabbit anti-GSDMD monoclonal antibody could react with porcine GSDMD protein in PK-15 cells by immunoblotting assays (Xu et al. 2023). However, they did not analyze whether the monoclonal antibody could react with porcine alveolar macrophage 3D4/21 cells, which were used in our present study. In order to find out the possible reasons for the unsatisfactory reactivity of commercial GSDMD antibodies, we aligned the amino acid sequence homology of GSDMD proteins among different animal species, including *Mus musculus*, *Homo sapiens*, and *Sus scrofa*, and found that the amino acid homology of porcine GSDMD protein (GenBank accession no. XP_020946165.1) with human (NP_079012) and murine GSDMD (NP081236.1) proteins is 64.5% and 56.7%, respectively (Supplemental Fig. S5). Moreover, we have taken a step further by determining whether there exist common conserved antigenic epitopes between human, mouse, and porcine GSDMD proteins (Supplemental Fig. S4). It should be pointed out that although the number of sequences of human, mouse, and pig GSDMD proteins in the current GenBank database is very limited, our results indicated that there are very rare conservative regions in the GSDMD protein of different species (Supplemental Fig. S4). This indicates that even if there exist common conserved antigenic epitopes, the number of them will be very rare. Therefore, human and murine GSDMD protein-specific antibodies are not suitable for the detection of pGSDMD protein and the study of pyroptosis in pigs in vivo and in vitro. Due to the lack of effective pGSDMD-specific antibodies, there have been relatively few studies on cell pyroptosis in pigs or pig-derived cells. So far, only a few swine pathogenic viruses have been reported to trigger pyroptosis after infection. For instance, classical swine fever virus (CSFV) was reported to be able to activate pyroptosis in the peripheral lymphoid organs of CSFV-infected pigs (Yuan et al. 2018). Porcine reproductive and respiratory syndrome virus was demonstrated to induce GSDMD-driven pyroptosis in porcine alveolar macrophages through NLRP3 inflammasome activation (He et al. 2022). Senecavirus A (SVA) was proved to induce both caspase-dependent and caspase-independent pyroptosis in SK6 cells, and the 3C^pro^ of SVA was further found to cleave pGSDMD protein at non-canonical cleavage sites Q193 and Q277 through its protease activity (Wen et al. 2021). ASFV cysteine protease S273R was discovered to inhibit pyroptosis by cleaving GSDMD at a non-canonical site (Zhao et al. 2022).

To provide an effective tool for investigating the structure and biological functions of pGSDMD protein, as well as the role of cell pyroptosis in pig growth and development, immune responses, and combating pathogen infections, we decided to prepare mAbs that can specifically recognize porcine GSDMD protein. To this end, we first predicted the important parameters of pGSDMD protein, such as antigenicity, transmembrane region, hydrophobicity, and protein secondary structure, and discovered that pGSDMD protein does not contain transmembrane and inside regions, and the epitopes are rich and evenly distributed (Fig. 1b, c), indicating that the pGSDMD protein has good antigenicity. Hence, we expressed the full-length pGSDMD protein using a prokaryotic expression system. Although we have attempted to optimize various inducible expression conditions, such as reducing IPTG concentration, lowering temperature, and shortening bacterial culture time, pGSDMD protein was still mainly expressed in the inclusion bodies. Consequently, we purified the recombinant pGSDMD protein using Ni–NTA resin under denaturing conditions. The purified pGSDMD protein was used to immunize BALB/c mice to generate mAbs by hybridoma technique. Finally, we successfully prepared five mAbs that can specifically react with pGSDMD protein and show no cross reactivity with human and murine GSDMD proteins.

To further verify whether the five mAbs are suitable for pyroptosis research in pig-derived cell lines, we selected three different cell lines from distinct species for western blot analysis using the prepared mAbs as the primary antibodies, along with treatment of the cells with LPS plus ATP as a positive control for pyroptosis activation (Shi et al. 2015). Our results indicated that all five mAbs can specifically recognize the full-length pGSDMD protein in the resting state of pig-derived cell line 3D4/21 and can also effectively recognize the N-terminal or C-terminal fragments produced when the cell line is in a state of pyroptosis activation. Undoubtedly, the mAbs we prepared have excellent specificity for studying the function and pyroptosis of pGSDMD in pigs, both in vivo and in vitro, and therefore has good commercial production prospects.

Furthermore, we identified the epitopes recognized by the five mAbs through construction of a series of truncated forms of pGSDMD protein. Amazingly, the epitopes of all five mAbs identified by serial truncations were completely distributed within the epitope range predicted by IEDB (Table 2 and Fig. 1c). In addition, using universal primers located within the framework region of monoclonal antibodies, PCR amplification combined with nucleotide sequencing and bioinformatic prediction, we ultimately obtained the sequences of the heavy- and light-chain CDR regions of all five mAbs, which provide valuable information for the direct expression of genetically engineered antibodies that can specifically recognize pGSDMD protein, such as chimeric antibodies, reconstructed antibodies, single chain antibodies, and anti-idiotypic antibodies.

Up to date, there have been few published papers on monoclonal or polyclonal antibodies raised against porcine GSDMD protein. Compared with the rabbit anti-pGSDMD polyclonal antibodies reported by Song et al. (2021), the five mAbs we prepared have better specificity, clear antigenic epitopes, and high homogeneity and can be produced in unlimited quantities. In addition, although the five mAbs we prepared and those prepared by Song et al. (2022) were all able to recognize the full-length pGSDMD protein, four (15H6, 19H3, 23H10, and 27A10) out of our five mAbs were able to recognize the N-terminal active fragment of pGSDMD protein, and one (25E2) recognized its C-terminal fragment (Fig. 6a–e). In contrast, the mAb mentioned by Song et al. (2022) could only react with the C-terminal fragment of pGSDMD protein.

In conclusion, we successfully prepared five mAbs that are able to specifically recognize pGSDMD protein, identified the epitopes recognized by these mAbs, and determined their respective CDR sequences. These mAbs provide valuable tools for the research on the biological functions of pGSDMD and pyroptosis in pigs both in vivo and in vitro and have good commercial production prospects.

## Supplementary Information

Below is the link to the electronic supplementary material.

Supplementary file1 (PDF 1106 KB)

## Data Availability

All experimental data related to this paper are either in the main text or in the supplementary materials.
