# Supplementary material for: Preparation and characterization of monoclonal antibodies against porcine gasdermin D protein
Source: Appl Microbiol Biotechnol. 2024 Jan 25;108:173. doi: 10.1007/s00253-023-12938-x (PMC10808365; doi:10.1007/s00253-023-12938-x)
Supplement: Supplementary file 1 [file 253_2023_12938_MOESM1_ESM.pdf]

Applied Microbiology and Biotechnology

Supplementary materials

Preparation and characterization of monoclonal antibodies against porcine gasdermin D protein

Minhui Yang[1,2], Xinna Ge[1,2], Lei Zhou[1,2], Xin Guo[1,2], Jun Han[1,2], Yongning Zhang[1,2*], Hanchun Yang[1,2]

[1] National Key Laboratory of Veterinary Public Health and Safety, College of Veterinary Medicine, China Agricultural University, Beijing 100193, People's Republic of China; [2] Key Laboratory of Animal Epidemiology of Ministry of Agriculture and Rural Affairs, College of Veterinary Medicine, China Agricultural University, Beijing 100193, People's Republic of China

* Corresponding authors at: National Key Laboratory of Veterinary Public Health and Safety, Key Laboratory of Animal Epidemiology of Ministry of Agriculture and Rural Affairs, College of Veterinary Medicine, China Agricultural University; No. 2 Yuanmingyuan West Road, Haidian District, Beijing 100193, People's Republic of China.

E-mail: zhangyongning@cau.edu.cn (Y. Zhang); Tel/fax: +86 10 62734924

ORCID：https://orcid.org/0000-0002-0134-2761

Supplemental Table S1 Primers used for constructing the eukaryotic expression plasmids pCMV-pGSDMD-FL, pCMV-pGSDMD-NT, and pCMV-pGSDMD-CT

Primer name	Primer sequence (5’-3’) [a]
pGSDMD-FL-F	TCCAAGCTTCTGCAGGAATTCATGGCATCAGCCTTTGAGAGG
pGSDMD-FL-R	CCCACTAGTTCTAGACTCGAG**AGCGTAATCTGGAACATCGTATGGGTA**GCAGAGCTGGCTGAGCC
pGSDMD-NT-F	TCCAAGCTTCTGCAGGAATTCATGGCATCAGCCTTTGAGAGG
pGSDMD-NT-R	CCCACTAGTTCTAGACTCGAGCTAGTCTGACTGGAACTTCAGGTGC
pGSDMD-CT-F	CGCGGGCCCAGGCCCGAATTCGGGCCCGCGGAGGACCAG
pGSDMD-CT-R	ATGGGTAGCCGGTACCTCGAGGCAGAGCTGGCTGAGCC

[a] Restriction enzyme sites are underlined. The encoding sequence of hemagglutinin (HA) tag is labeled in bold font.

Supplemental File S1 The encoding gene of pGSDMD protein after codon optimization

ATGGCTTCAGCATTTGAAAGGGTAGTTAAATCCGTGGTTCGTGAACTGGATCATGGTCGTGA
GCTGACCCCGGTTAAAAGCCTACAAACTTCAGATCGTTTTCAGCCGTACTGCCTGCTGGGC
CGCAAGCCGAGCTCATCCTGGTTCTGGCGTCCGCGTTATACCTGTGTTGACCTGAGCATTTG
GGACATCCTGGAGCCGAGCGCGCCTGAGCCGGCTGTCGAGCGCGGTGGTCCGTTTTATTTC
CACGATACCATGGATGGTCAGCTGCAGGGCCAAGTGGAACTGGCGGCACCGGGTCAAGGC
AAATTCAGCGGTGGTGCTGCGGTGTCTGGCTCCTCCTCTGCTTCCATGAATGTTTGTACCTT
ACGCGTGGCGCCGAACACCTGGGATGCAATGCATCTAGAACGCCGTCTGCGTCAGCCGGA
ACACAAGGTGTTACAGCAGCTCCGTTCTCGCGGAAACGATGTGTTTGTTGTTACGGAAGTA
CTGCAAACCCAGAAAGAAGTAGAGGTTACGCGTACCCATAAGCAAGAGGGTTCGGGTCAG
TTTGCCTTGCCGGGTGCGGTGAGCTTGCAAGGCCAGGGTCAAGGCCACCTGTCCCGTAAG
AAAACCGTTACCATTCCGTCTGGTAGCGTTATTGCATTTCGTGTGGCGCAACTGGTTATTGG
TTCCGACTGGGACATCCTGCTGTTCCCGGATAAAAAGCAGCGTACCTTTCGCCCACTGCGT
GAAGGTCACAGCGCGAGCCACGGCGCGGATGGCCAACCGCCGCAGTTCAGCCGCTTGGTT
TCTGGGATGAGCTTTCCGAGCGAGCACCTGAAGTTCCAAAGCGACGGCCCGGCGGAAGAT
CAGCTGGTGACCACCGAAGACTTCCAGGGCTTGCAAGCGGAAGTCGACGCGTGGGCGGCA

GGTCTGGAGGCCCTTAGCAGAGAACCGTGTCGTCAGTTGCTGGGTGGCCTCGGTCAGGTCCTTCAAGACGAACCAGCACTCCAAGCCTTAGAGGACAGCCTGGAACAGGGCCTGTGCGGTGGCTTGGTGGAGCCGCAAGACGGCCCGGCAGGCGCTATGCTGGAGTGCCTGGTCCTTGCGTGCAGACGTCTGGAGCGTGAGGTGGCCGGCCCAATCTTCTACCTCGTCCAGGCTTTGGCTGTGCTGAACGAGACTCAACATGTTCTGCTGGCGAAAGTGTTGGAGACTGGTGCTTTGAGCGGCGCGTTGACCCTGGTAGGTTCACTGCTGGAGCAGTCGGCGCCGTGGCAGGAGCGCCGGGCAGTTTCTCTTCCGCCAACGCTCCTGGGTTCGAGCTGGGGTAGCGAAGCTCCGATCTGGGCGCTCTTGGAGGAATGCGGTCTGGAACCGCAGGTGGGCACCGCACAGGTTTGCTGGGAACCGGAAGCGCAATCTTGTACGTGCGCATTATACGCCTGCCTGGCACTGTTGCTACGTCTGAGCCAGCTGTGC

Supplemental Table S2 Primers used for amplifying the heavy- and light-chain variable regions of the five mouse anti-pGSDMD mAbs

Primer name	Primer sequence (5'-3')
VH-F1	TGAGGAGACGGTGACCGTGGTCCCTTGGCCCC
VH-R1	AGGTSMARCTGCAGSAGTCWGG
VH-F2	GAGGTGAAGCTTCTCGAGTCTGG
VH-R2	TGAGGAGACGGTGACCATGGTCCC
VL-F2	AGGAGACAGACACACTCCTGCTAT
VL-R2	CCCAAGCTTACTGGATGGTGGGAAATGGA

Supplemental Table S3 Primers used for constructing the prokaryotic plasmids expressing GST-tagged truncated forms of pGSDMD protein

Round	Truncation	Primer	Primer sequence (5'-3') [a]
Round 1	1-279aa	1-104aa-F	CCCCTGGGATCCCCGGAATTCATGGCTTCAGCATTTGAAAGGG
		1-104aa-R	GTCACGATGCGGCCGCTCGAGCTAGAATTTGCCTTGACCCGG
		43-153aa-F	CCCCTGGGATCCCCGGAATTCATGAAGCCGAGCTCATCCTG
		43-153aa-R	GTCACGATGCGGCCGCTCGAGCTAGCGAGAACGGAGCTGCT
		126-235aa-F	CCCCTGGGATCCCCGGAATTCATGGCGCCGAACACCTGG

		126-235aa-R	GTCACGATGCGGCCGCTCGAGCTATTTATCCGGGAACAGCAGG
		180-279aa-F	CCCCTGGGATCCCCGGAATTCATGGGTTCGGGTCAGTTTGC
		180-279aa-R	GTCACGATGCGGCCGCTCGAGCTAGTCGCTTTGGAACTTCAGGTG
	280-488aa	280-337aa-F	CCCCTGGGATCCCCGGAATTCATGGGCCCGGCGGAAGAT
		280-337aa-R	GTCACGATGCGGCCGCTCGAGCTAGCTGTCCTCTAAGGCTTGGA
		300-375aa-F	CCCCTGGGATCCCCGGAATTCATGGACGCGTGGGCGGCA
		300-375aa-R	GTCACGATGCGGCCGCTCGAGCTATGGGCCGGCCACCTC
		350-445aa-F	CCCCTGGGATCCCCGGAATTCATGCAAGACGGCCCGGCA
		350-445aa-R	GTCACGATGCGGCCGCTCGAGCTACGCCCAGATCGGAGCT
		401-488aa-F	CCCCTGGGATCCCCGGAATTCATGGGTGCTTTGAGCGGC
		401-488aa-R	GTCACGATGCGGCCGCTCGAGCTAGCACAGCTGGCTCAGACG
Round 2	1-50aa	1-20aa-F	GGGATCCCCGGAATTCATGGCTTCAGCATTTGAAAGGGT
		1-20aa-R	GATGCGGCCGCTCGAGACGACCATGATCCAGTTCACG
		9-29aa-F	GGGATCCCCGGAATTCGTTAAATCCGTGGTTCGTGAACTGGA
		9-29aa-R	GATGCGGCCGCTCGAGTTGTAGGCTTTTAACCGGGGTCAGCTCAC
		23-40aa-F	GGGATCCCCGGAATTCACCCCGGTTAAAAGCCTACAAAC
		23-40aa-R	GATGCGGCCGCTCGAGCAGCAGGCAGTACGG
		32-50aa-F	GGGATCCCCGGAATTCGATCGTTTTCAGCCGTACTGCCTGCT
		32-50aa-R	GATGCGGCCGCTCGAGCCAGAACCAGGATGAGCT
	43-104aa	43-63aa-F	GGGATCCCCGGAATTCAAGCCGAGCTCATCCTGG
		43-63aa-R	GATGCGGCCGCTCGAGGTCCCAAATGCTCAGGTCAACACAGGT
		53-79aa-F	GGGATCCCCGGAATTCCGTTATACCTGTGTTGACCTGAGCATTTGG
		53-79aa-R	GATGCGGCCGCTCGAGCGGACCACCGCGCT
		70-93aa-F	GGGATCCCCGGAATTCCCTGAGCCGGCTGTCG
		70-93aa-R	GATGCGGCCGCTCGAGTTGGCCCTGCAGCTGAC
		83-104aa-F	GGGATCCCCGGAATTCCACGATACCATGGATGGT
		83-104aa-R	GATGCGGCCGCTCGAGGAATTTGCCTTGACCCGGTG
	230-279aa	230-249aa-F	GGGATCCCCGGAATTCCTGCTGTTCCCGGATAAAAAG
		230-249aa-R	GATGCGGCCGCTCGAGCGCGCTGTGACCTTCACGCAGT

		240-258aa-F	GGGATCCCCGGAATTCTTTCGCCCACTGCGTG
		240-258aa-R	GATGCGGCCGCTCGAGCGGCGGTTGGCCATCC
		251-268aa-F	GGGATCCCCGGAATTCCACGGCGCGGATG
		251-268aa-R	GATGCGGCCGCTCGAGGCTCATCCCAGAAACCAAGCG
		260-279aa-F	GGGATCCCCGGAATTCTTCAGCCGCTTGGTTTCTGG
		260-279aa-R	GATGCGGCCGCTCGAGGTCGCTTTGGAACTTCAGGTGCT
		280-410aa-F	GGGATCCCCGGAATTCGGCCCGGCGGAAG
		280-410aa-R	GATGCGGCCGCTCGAGTACCAGGGTCAACGCGCCGC
		280-420aa-F	GGGATCCCCGGAATTCGGCCCGGCGGAAG
		280-420aa-R	GATGCGGCCGCTCGAGCCACGGCGCCGAC
		280-430aa-F	GGGATCCCCGGAATTCGGCCCGGCGGAAG
		280-430aa-R	GATGCGGCCGCTCGAGTGGCGGAAGAGAAACTGCCC
		280-440aa-F	GGGATCCCCGGAATTCGGCCCGGCGGAAG
		280-440aa-R	GATGCGGCCGCTCGAGTTCGCTACCCCAGCTCGAACCCA
Round 3	24-37aa	24-32aa-F	GGGATCCCCGGAATTCCCGGTTAAAAGCCTACAAACTTCAGATCTCGAGCGGCCGCATC
		24-32aa-R	GATGCGGCCGCTCGAGATCTGAAGTTTGTAGGCTTTTAACCGGGAATTCCGGGGATCCC
		26-34aa-F	GGGATCCCCGGAATTCAAAAGCCTACAAACTTCAGATCGTTTTCTCGAGCGGCCGCATC
		26-34aa-R	GATGCGGCCGCTCGAGAAAACGATCTGAAGTTTGTAGGCTTTTGAATTCCGGGGATCCC
		28-37aa-F	GGGATCCCCGGAATTCCTACAAACTTCAGATCGTTTTCAGCCGTACCTCGAGCGGCCGCATC
		28-37aa-R	GATGCGGCCGCTCGAGGTACGGCTGAAAACGATCTGAAGTTTGTAGGAATTCCGGGGATCCC
	74-88aa	74-82aa-F	GGGATCCCCGGAATTCGTCGAGCGCGGTGGTCCGTTTTATTTCCTCGAGCGGCCGCATC
		74-82aa-R	GATGCGGCCGCTCGAGGAAATAAAACGGACCACCGCGCTCGACG

		AATTCCGGGGATCCC
	76-84aa-F	GGGATCCCCGGAATTCCGCGGTGGTCCGTTTTATTTCCACGATCTCGAGCGGCCGCATC
	76-84aa-R	GATGCGGCCGCTCGAGATCGTGGAAATAAAACGGACCACCGCGGAATTCCGGGGATCCC
	78-86aa-F	GGGATCCCCGGAATTCGGTCCGTTTTATTTCCACGATACCATGCTCGAGCGGCCGCATC
	78-86aa-R	GATGCGGCCGCTCGAGCATGGTATCGTGGAAATAAAACGGACCGAATTCCGGGGATCCC
	80-88aa-F	GGGATCCCCGGAATTCTTTTATTTCCACGATACCATGGATGGTCTCGAGCGGCCGCATC
	80-88aa-R	GATGCGGCCGCTCGAGACCATCCATGGTATCGTGGAAATAAAAGAATTCCGGGGATCCC
253-265aa	253-260aa-F	GGGATCCCCGGAATTCGCGGATGGCCAACCGCCGCAGTTCCTCGAGCGGCCGCATC
	253-260aa-R	GATGCGGCCGCTCGAGGAACTGCGGCGGTTGGCCATCCGCGAATTCCGGGGATCCC
	255-262aa-F	GGGATCCCCGGAATTCGGCCAACCGCCGCAGTTCAGCCGCCTCGAGCGGCCGCATC
	255-262aa-R	GATGCGGCCGCTCGAGGCGGCTGAACTGCGGCGGTTGGCCGAATTCCGGGGATCCC
	257-264aa-F	GGGATCCCCGGAATTCCCGCCGCAGTTCAGCCGCTTGGTTCTCGAGCGGCCGCATC
	257-264aa-R	GATGCGGCCGCTCGAGAACCAAGCGGCTGAACTGCGGCGGGAATTCCGGGGATCCC
	259-265aa-F	GGGATCCCCGGAATTCCAGTTCAGCCGCTTGGTTTCTCTCGAGCGGCCGCATC
	259-265aa-R	GATGCGGCCGCTCGAGAGAAACCAAGCGGCTGAACTGGAATTCCGGGGATCCC

425-440aa	425-433aa-F	GGGATCCCCGGAATTCGCAGTTTCTCTTCCGCCAACGCTCCTGCTCGAGCGGCCGCATC
	425-433aa-R	GATGCGGCCGCTCGAGCAGGAGCGTTGGCGGAAGAGAAACTGCGAATTCCGGGGATCCC
	427-435aa-F	GGGATCCCCGGAATTCTCTCTTCCGCCAACGCTCCTGGGTTCGCTCGAGCGGCCGCATC
	427-435aa-R	GATGCGGCCGCTCGAGCGAACCCAGGAGCGTTGGCGGAAGAGAGAATTCCGGGGATCCC
	429-437aa-F	GGGATCCCCGGAATTCCCGCCAACGCTCCTGGGTTCGAGCTGGCTCGAGCGGCCGCATC
	429-437aa-R	GATGCGGCCGCTCGAGCCAGCTCGAACCCAGGAGCGTTGGCGGGAATTCCGGGGATCCC
	431-440aa-F	GGGATCCCCGGAATTCACGCTCCTGGGTTCGAGCTGGGGTAGCGAACTCGAGCGGCCGCATC
	431-440aa-R	GATGCGGCCGCTCGAGTTCGCTACCCCAGCTCGAACCCAGGAGCGTGAATTCCGGGGATCCC

[a] Restriction sites are underlined.

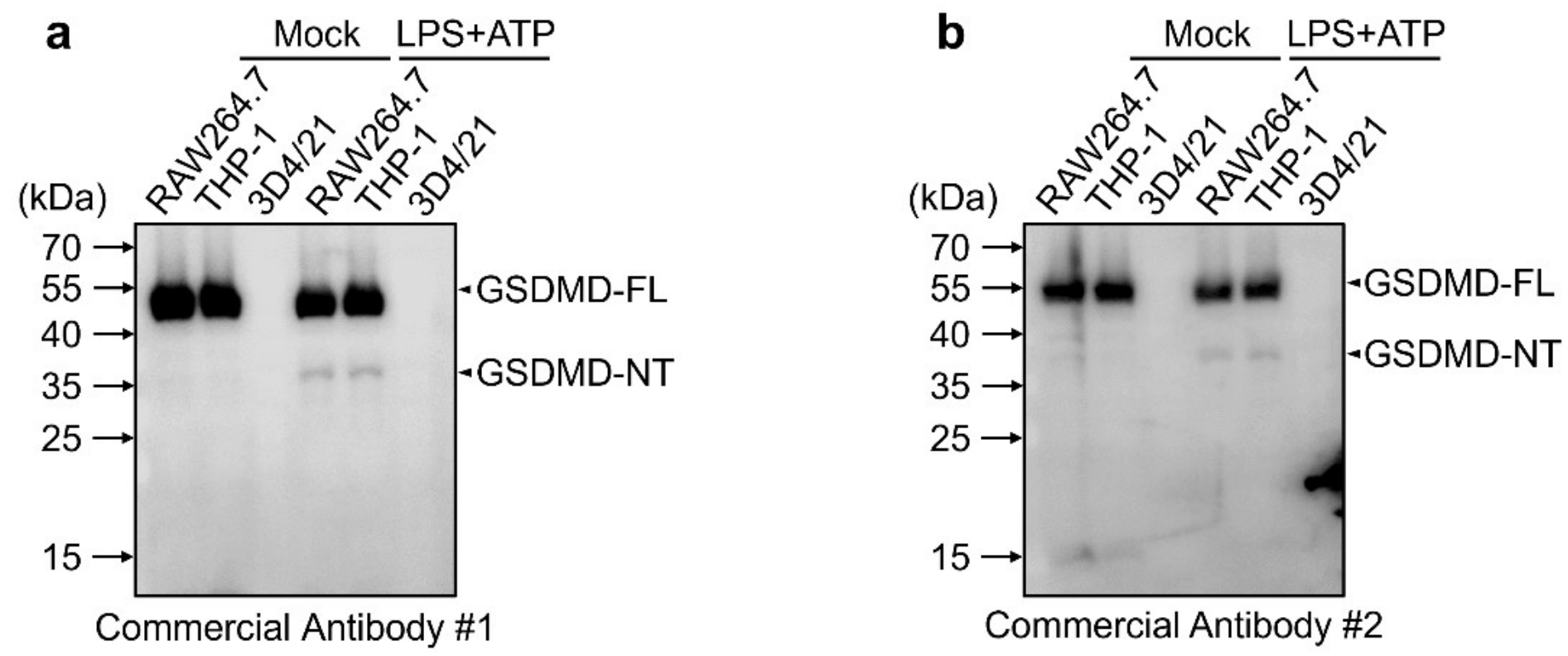

Supplemental Fig. S1 Western blot analysis of endogenous GSDMD proteins in three cell lines from different species using two commercially available rabbit anti-GSDMD polyclonal antibodies (#1: Proteintech, 20770-1-AP; #2: ABclonal, A20197).

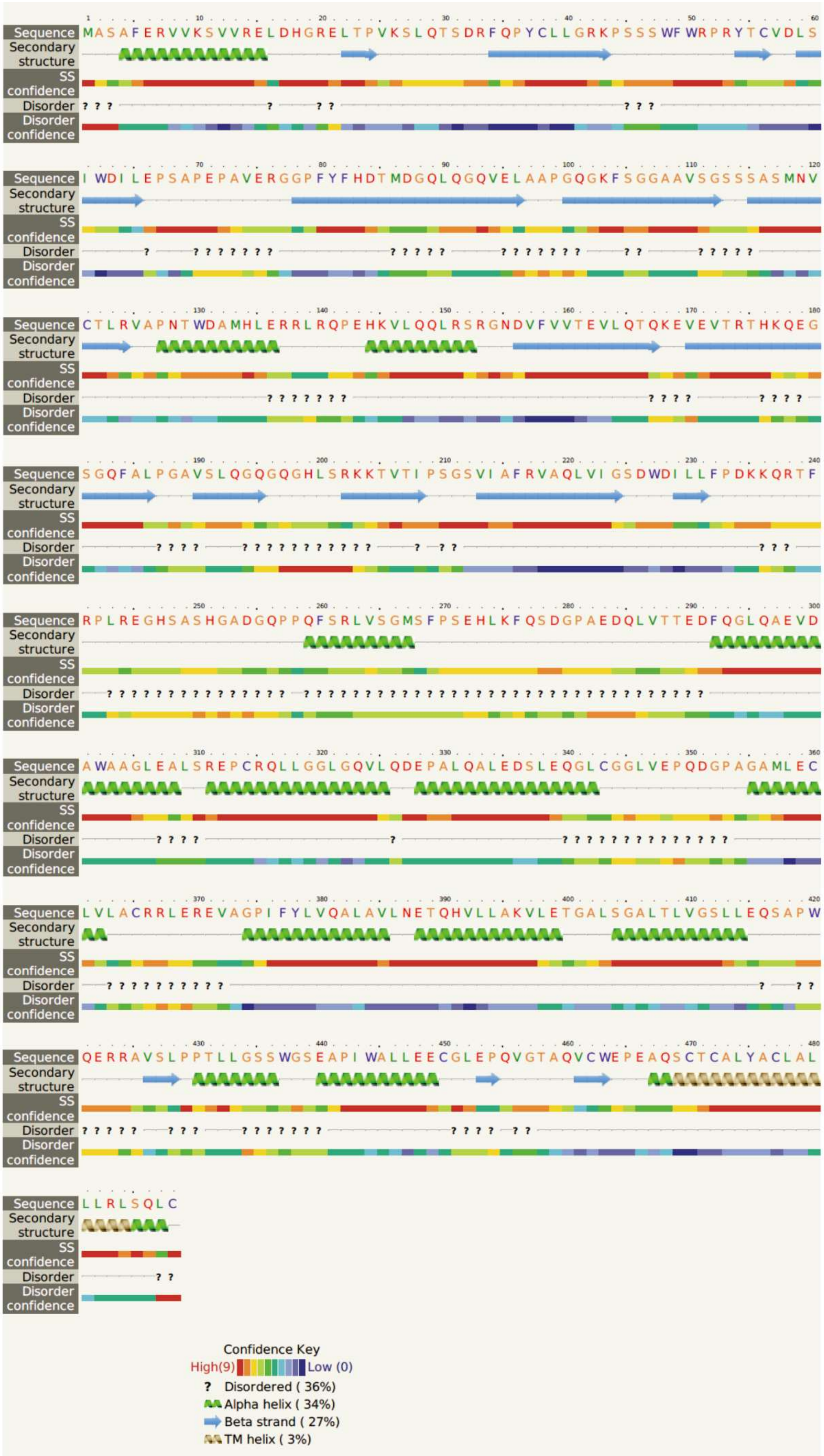
Sequence
Secondary structure
SS confidence
Disorder
Disorder confidence
MASAFERVVKSVVRELDHGRELTPVKSLQTSDRFQPYCLLGRKPSSSWFWRPRYTCVDLS
IWDILEPSAPEPAVERGGPFYFHDTMDGQLQGQVELAAPGQGKFSGGAAVSGSSSASMNV
CTLRVAPNTWDAMHLERRLRQPEHKVLQQLRSRGNDVFVVTEVLQTQKEVEVTRTHKQEG
SGQFALPGAVSLQGQGQGHLSRKKTVTIPSGSVIAFRVAQLVIGSDWDILLFPDKKQRTF
RPLREGHSASHGADGQPPQFSRLVSGMSFPSEHLKFQSDGPAEDQLVTTEDFQGLQAEVD
AWAAGLEALSREPCRQLLGGLGQVLQDEPALQALEDSLEQGLCGGLVEPQDGPAGAMLEC
LVLACRRLEREVAGPIFYLVQALAVLNETQHVLLAKVLETGALSGALTLVGSLLEQSAPW
QERRAVSLPPTLLGSSWGSEAPIWALLEECGLEPQVGTAQVCWEPEAQSCTCALYACLAL
LLRLSQLC
Confidence Key
High(9) Low (0)
? Disordered (36%)
Alpha helix (34%)
Beta strand (27%)
TM helix (3%)

Supplemental Fig. S2 Secondary structure prediction of pGSDMD protein using Phyre[2] server. The amino acid sequence of the full-length pGSDMD protein contains 34% α-helices, suggesting that the protein has high hydrophobicity and may be difficult to be expressed in soluble form.

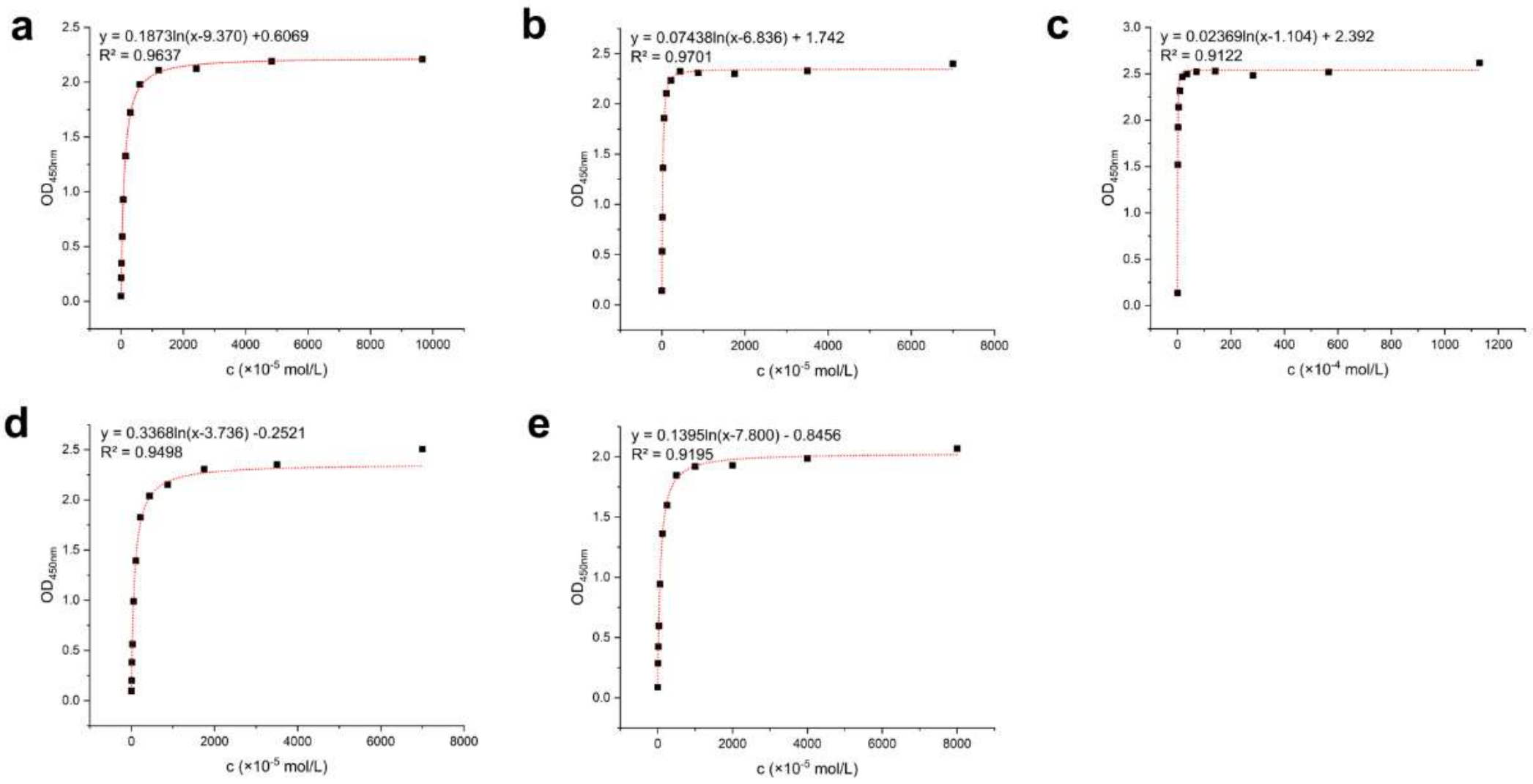

Supplemental Fig. S3 Affinity determination of the prepared mAbs specifically recognizing pGSDMD protein. (**a-e**) The fitted curve of mAbs 15H6, 19H3, 23H10, 27A10, and 25E2, respectively.

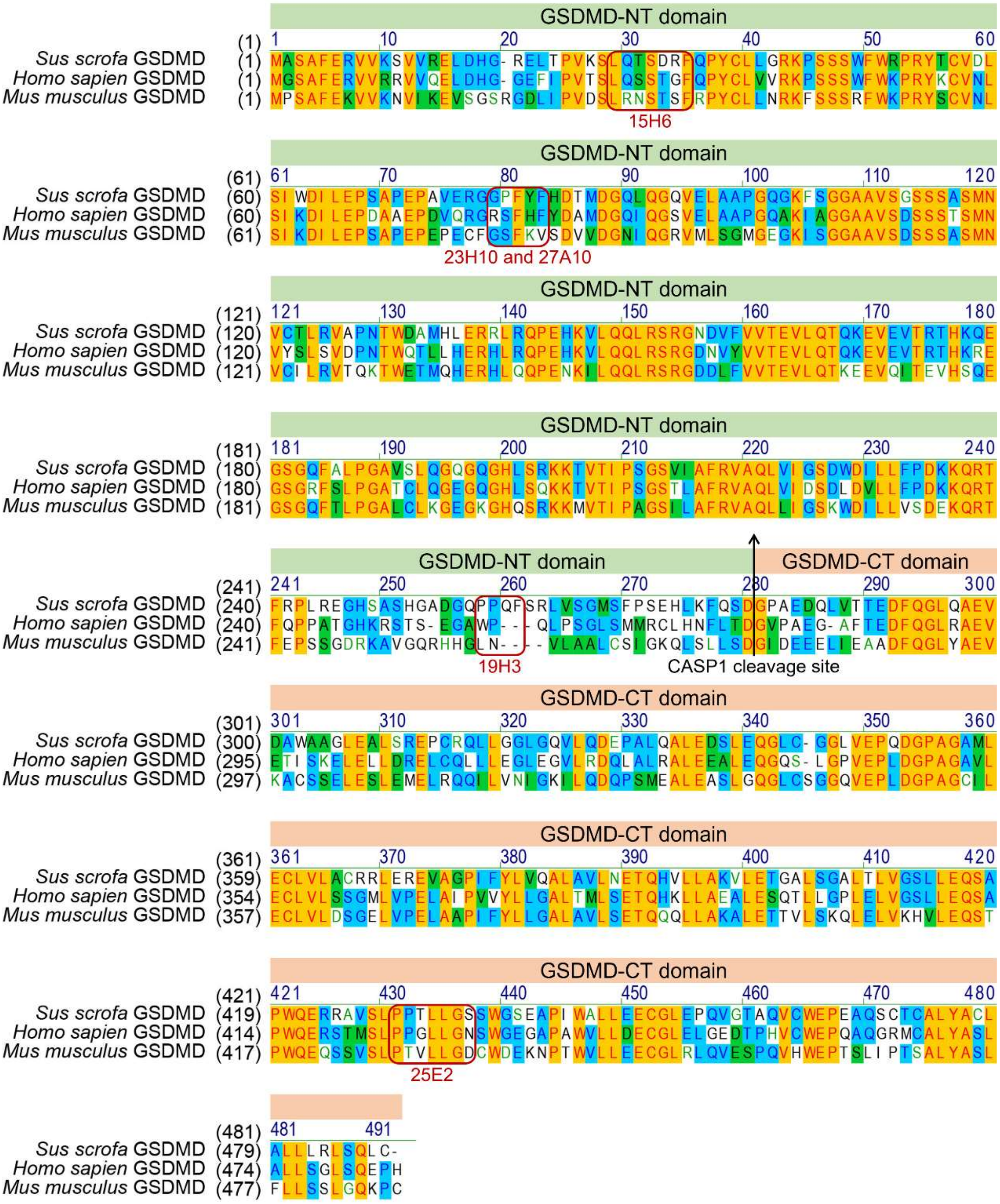

Supplemental Fig. S4 Conservative analysis of antigenic epitopes recognized by the five monoclonal antibodies in porcine, human, and mouse GSDMD proteins. The GenBank accession numbers for GSDMD proteins *of Sus scrofa*, *Homo sapiens*, and *Mus musculus* GSDMDs are XP_020946165.1, NP_001159709.1, and NP_081236.1, respectively. The identified antigenic epitopes are labeled with boxes.

Percent Identity

Divergence	1	2	3	4	5	6	7		
1		64.5	56.7	56.4	64.3	74.1	73.5	1	Sus_scrofa-XP_020946165.1
2	46.7		55.8	56.4	97.7	63.0	60.3	2	Homo_sapiens-NP_001159709.1
3	60.6	61.6		81.1	55.4	56.0	54.4	3	Mus_musculus-NP_081236.1
4	59.3	58.9	19.2		56.0	55.3	53.4	4	Rattus_norvegicus-NP_001387923.1
5	47.0	2.3	62.5	59.8		62.4	59.7	5	Pan_troglodytes-XP_009454389.3
6	30.3	47.7	59.7	59.7	48.8		88.3	6	Bos_taurus-NP_001346905.1
7	31.5	52.7	63.7	64.2	53.9	11.6		7	Capra_hircus-ALN66870.1
	1	2	3	4	5	6	7		

Supplemental Fig. S5 Homology comparison of amino acid sequences of GSDMD proteins from different species. The species and GenBank accession numbers are marked in the figure.
